# Outcome of endovascular stroke therapy in a large mandatory stroke-registry

**DOI:** 10.1186/s42466-023-00287-z

**Published:** 2023-12-21

**Authors:** Sonja Hyrenbach, Susanne Rode, Martin Schabet, Michael Daffertshofer, Karin Schoser, Stephan Neumaier, Peter A. Ringleb

**Affiliations:** 1 Stroke Working Group, Office for Quality Assurance in Hospitals (QiG BW), Stuttgart, Germany; 2 Department of Neurology, Ruprecht Karls University Heidelberg, Im Neuenheimer Feld 400, 69120 Heidelberg, Germany

**Keywords:** Stroke, Thrombectomy, Thrombolysis, Health care research

## Abstract

**Background:**

Endovascular stroke treatment (EST) has become the standard treatment for patients with stroke due to large vessel occlusion, especially in earlier time windows. Only few data from population-based registries on effectiveness of EST have been published.

**Methods:**

Baden–Wuerttemberg is the third largest state in Germany in terms of area and population and has a structured stroke concept since 1998 which includes mandatory collection of quality assurance data. In 2018 and 2019, 3820 of 39,168 ischemic stroke patients (9.8%) were treated by EST (age median 78 y, NIHSS median 14). We analyzed the clinical outcome of these patients determined with the modified Rankin Scale (mRS) at discharge from the hospital or with the initiation of palliative therapy using logistic regression analysis with adjustment for the mRS at admission, additive IVT, age, and NIHSS.

**Results:**

The probability of an excellent clinical outcome (mRS 0 or 1 at discharge) and for a good clinical outcome (mRS 0–2) were significantly higher in EST-patients (odds-ratio (OR) 1.27; 95% confidence interval (95% CI) 1.13–1.43, and OR of 1.15 (95% CI 1.04–1.28). Also, the regression model showed an advantage for EST-patients with less frequent ‘decision for palliative care’ (OR 0.87; 95% CI 0.78–0.98). Sensitivity analysis adjusting for intracranial vessel occlusion as further factor showed similar results.

**Conclusion:**

Our data suggest that EST can be of benefit also for an area-wide unselected stroke population, in a large German federal state with sometimes long distance to the next thrombectomy center.

## Introduction

Endovascular stroke therapy (EST) has fundamentally changed the treatment of acute ischemic stroke patients in recent years. Several randomized clinical trials (RCT) showed a clinically highly relevant improvement of outcome after EST in selected patients with intracranial large vessel occlusion [1–6]. Based on an individual patient metaanalysis, including the data from 1.287 patients from five RCTs the, EST including intravenous thrombolysis in 87% of cases led to significantly reduced disability (modified Rankin Scale 0 to 2 at 90 days; adjusted common odds ratio 2.71, 95% CI 20.7 to 3.55) compared with prior standard treatment [7]. Almost any international guideline now recommends EST as standard of care in suitable patients [8, 9]. Therefore, over the last few years, EST has become an integral part of routine standard of care of acute stroke patients.

However, data on EST in clinical practice still are sparse. It is still not finally answered, whether the benefit of EST is also present in an unselected cohort. RCTs have enrolled highly selected patients with a huge likelihood to benefit from vessel recanalization for a proof of concept without increased risk. Patients were selected based on the type of vessel occlusion (i.e. only anterior circulation occlusion) and time window (i.e. treatment within 6 h.). In addition, those trials were done in stroke centers with al lot of experience in treating patients with severe stroke including endovascular stroke treatment. It remains unclear whether structural differences between regions and their hospitals as well as heterogeneity regarding the expertise of interventionalists significantly influences the overall beneficial effect of EST. It is also unclear if the EST-effect can already be detected in the early stage, e.g. at discharge from the acute hospital. Therefore, we analyzed the data of the comprehensive stroke registry concerning Baden–Wuerttemberg, the third largest state in Germany in terms of area and population, with respect to mRS at discharge and the decision to adopt a palliative approach during the course of in-hospital stroke treatment.

## Methods

Baden–Wuerttemberg (BW) is the third largest German federal state by area, accounting for 10% of Germany's total area at 35,747.82 km^2^, and around 11 million inhabitants, 13.4% of the German population (31.12.2020) [10]. In Baden Württemberg a structured concept for stroke patients has been in place since 1998. Accordingly, all stroke patients should be treated close to home in a stroke unit, which has three levels, to ensure early diagnosis and treatment. Local stroke units, mostly run under the direction of departments of internal medicine, offer 24/7 CT including CTA and intravenous thrombolysis (IVT) 24/7. Regional stroke units provide 24/7 neuroradiology, 24/7 CT with CT angiography, and MRI. Some of these provide EST, mostly during routine working hours (“sunshine thrombectomy”). Stroke centers provide the whole diagnostics, IVT and EST 24/7, and additionally have neurosurgery and vascular surgery in the same hospital. Cooperation between stroke units of different levels have been established to ensure the care of complex stroke patients. As part of a mandatory external quality assurance procedure, any adult patient with an acute ischemic or hemorrhagic stroke and none of the exclusion diagnoses (e.g., traumatic brain injury or brain tumor) is included in the BW-stroke registry. If a patient is treated at multiple sites, a separate record is generated at each site. A comprehensive description of the BW-stroke registry is provided elsewhere [11].

We performed a retrospective database study of the prospectively collected BW-stroke registry data. In 2018 and 2019 overall 83,237 data sets were submitted. For this analysis we included patients with complete datasets and ischemic stroke (ICD-10 I63.*) and excluded patients where time between symptom onset and admission exceeded 24 h, patients with inhouse strokes, and patients who received palliative care from the start. In addition, if patients were immediately transferred for recanalizing therapy to another hospital, only the record from the second hospital was included in this analysis, to prevent double inclusion of data.

**Fig. 1:**
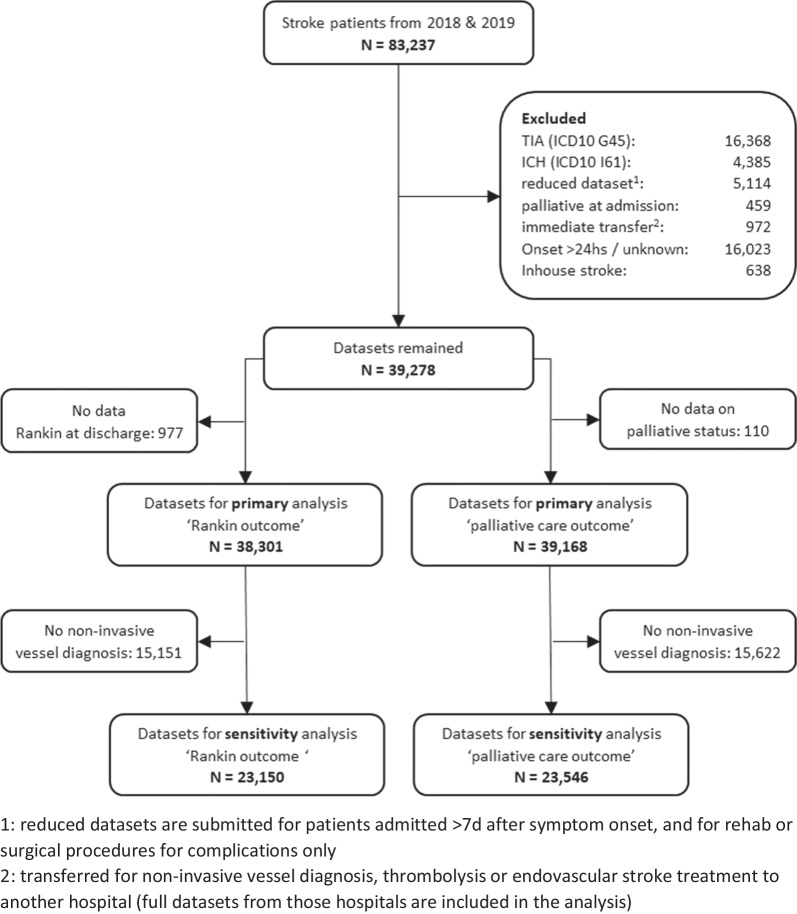
Flowchart describing the composition of the cohorts studied, in particular showing the reasons why datasets were excluded

The endpoints of interest were (1) the value on the modified Rankin-scale at discharge and (2a) in-hospital mortality and (2b) decision to receive palliative care over the course of the in-hospital treatment. Datasets with missing outcome information had be excluded from the analysis as well. Figure 1 shows the composition of our cohort and in particular how many patients were excluded and for what reason. Overall, 38,301 datasets remained for the analysis of the clinical outcome, and 39,168 datasets for the analysis of decision for palliative care.

SAS V9.4. was used for statistical analysis. As no correlation between the continuous variables age, NIHSS, and mRS at admission were found, the thrombectomy and no thrombectomy groups were first tested for differences in outcome parameters using t-tests. If there was a significant difference, variables were included in a multivariate analysis. Odds ratios (OR) were calculated by multiple logistic regression (stepwise selection) with adjustment for age, NIHSS, mRS at admission and iv thrombolysis. The computer algorithm does not provide the OR for variables rejected during the stepwise selection. Age was categorized into 18–64, 65–80, and above 80 years, and the NIHSS was categorized as 0, 1–5, 6–9, and ≥ 10 points. We used categories instead of continues values because of the non-linear importance of age and NIHSS for stroke outcome. For sensitivity analysis, the cohort was additionally restricted to cases with vascular imaging, and risk adjustment was added by occlusion of intracranial vessels.

## Results

According to the Baden–Wuerttemberg stroke registry, 3820 acute stroke patients were treated with EST in 2018 and 2019. This translates into a country wide proportion of 9.8% EST-treatment in patients with ischemic stroke. Baseline data of the patients used for the analysis of the endpoint ‘modified Rankin at discharge’ are shown in Table 1. Of note, median NIHSS of the EST-patients was 14 (Interquartile-range (IQR) 10) and the median age 78 years (IQR 16). Almost 55% of the EST-patients and 22% of the non-EST-patients received IVT. EST treatment was done in 21 different hospitals. Excellent clinical outcome (mRS 0–1 at discharge) was reached by 41.6% of the patients, and good clinical outcome (mRS 0–2) by 61.9%; in-hospital mortality was 6.5% and in 6.8% a decision for palliative care was made. Table 2 shows the proportion of those endpoints for different recanalizing treatment regimes. The correlation analysis confirmed the independence of the variables age, NIH-SS and mRS at admission (also see methods), which therefore were included into the logistic regression model. After adjusting for mRS at admission, IVT, age, and NIHSS the regression analysis (Table 3a) revealed that the probability of an excellent clinical outcome at discharge (mRS 0 or 1) was significantly higher in EST-patients (odds-ratio (OR) 1.27; 95%confidence interval (95% CI) 1.13–1.43; p < 0.0001). The same was shown for good clinical outcome (mRS 0 to 2) with an odds-ratio of 1.15 (95% CI 1.04–1.28; p = 0.0065). When the analysis was restricted to patients with non-invasive vascular imaging and large-vessel occlusions added to the risk adjustment (Table 3b), the positive effects were even stronger: The OR for an excellent clinical outcome (mRS 0 or 1) at discharge after EST was 1.572 (95% CI 1.34–1.85; p < 0.0001), and 1.57 (95% CI 1.36–1.81; p < 0.0001) for good clinical outcome (mRS 0 to 2). The in-hospital mortality was lower in EST-patients with an OR of 0.69 (95% CI 0.59–0.79; p < 0.0001).

**Table 1:** Baseline data of patients with ischemic stroke (I63.*) and data on mRS at discharge, with a time window</=24 h in the years 2018 and 2019 with respect to endovascular stroke treatment (N = 38,301)

	Endovascular stroke treatment done	No endovascular stroke treatment
Number of patients	3745	34,556
Female [n (%)]	1958 (52.3)	15,739 (45.6)
Age [Median (IQR)]	78 (16)	77 (16)
mRS admission [Median (IQR)]	5 (1)	3 (2)
NIHSS admission [Median (IQR)]	14 (10)	3 (5)
Non invasive vessel diagnosis [n (%)]	3745 (100)	21,248 (61.49)
Intracranial vessel occlusion [n (%)]	3745 (100)	3436 (9.94)
Intravenous thrombolysis [n (%)]	2059 (55.0)	7665 (22.2)
Time window</=5 h [n (%)]	2919 (77.9)	20,055 (58.0)
Diabetes mellitus [n (%)]	776 (20.7)*	8698 (25.2)
Hypertension [n (%)]	2940 (78.5)*	28,362 (82.1)
Atrial fibrillation [n (%)]	1804 (48.2) *	9819 (28.4)
Previously known	1222 (32.6)	7180 (20.8)
Newly diagnosed	582 (15.5)	2639 (7.6)

*Data missing from 2 patients; IQR: Interquartile-range

**Table 2:** Number and proportions of patients who reached different endpoints with respect to revascularization treatment (N = 38,301 for the mRS-endpoints and N = 39,168 for the endpoint ‘palliative care’)

Outcome	Intervention	n	Rate (%)
mRS 0–1	EST	667	1.74
	IVT	3609	9.42
	EST & IVT	421	1.1
	neither	11,225	29.31
mRS 0–2	EST	1136	2.97
	IVT	5502	14.37
	EST & IVT	706	1.84
	neither	16,373	42.74
mRS 6	EST	657	1.72
	IVT	783	2.04
	EST & IVT	342	0.89
	neither	722	1.89
Palliative	EST	637	1.63
	IVT	771	1.97
	EST & IVT	320	0.82
	neither	937	2.39

*mRS* modified Rankin score, *EST* endovascular stroke treatment, *IVT* intravenous thrombolysis

**Table 3:** Results of the logistic regression analysis for different endpoints modified Rankin-Scale (mRS) at discharge, in hospital mortality (mRS 6), and decision for palliative care during the hospital treatment. Given are odds-ratios and 95% confidence-intervals

Factor	mRS 0–1	mRS 0–2	mRS 6	Palliative care
*(a) Adjusted for mRS at admission, additive IVT, age, and NIHSS*				
EST	1.27 (1.13–1.43)	1.15 (1.04–1.28)	No effect	0.87 (0.78–0.98)
Age ≤ 65y	1.59 (1.49–1.71)	No effect	0.42 (0.35–0.51)	No effect
Age 65–80y	No effect	0.57 (0.53–0.62)	No effect	2.70 (2.19–3.33)
Age > 80y	0.65 (0.61–0.69)	0.35 (0.33–0.38)	1.90 (1.72–2.10)	5.84 (4.77–7.15)
NIHSS 1–5	0.64 (0.57–0.71)	0.66 (0.58–0.76)	No effect	No effect
NIHSS 6–9	0.39 (0.34–0.44)	0.38 (0.33–0.44)	1.65 (1.38–1.97)	1.86 (1.55–2.24)
NIHSS>/=10	0.28 (0.24–0.33)	0.28 (0.24–0.32)	5.00 (4.26–5.88)	6.30 (5.33–7.44)
IVT	2.53 (2.37–2.71)	2.39 (2.24–2.56)	0.68 (0.62–0.75)	0.62 (0.56–0.69)
*(b) Restricted to patients with non-invasive vessel diagnosis and adjusted also for intracranial vessel occlusion*				
EST	1.57 (1.34–1.85)	1.57 (1.36–1.81)	0.69 (0.59–0.79)	No effect
Age ≤ 65y	No effect	1.75 (1.59–1.93)	0.41 (0.33–0.51)	0.38 (0.32–0.49)
Age 65–80y	0.61 (0.56–0.66)	No effect	No effect	No effect
Age > 80y	0.38 (0.35–0.41)	0.61 (0.57–0.66)	2.04 (1.81–2.29)	2.15 (1.91–2.41)
NIHSS 1–5	0.59 (0.519–0.69)	0.64 (0.52–0.77)	No effect	No effect
NIHSS 6–9	0.39 (0.33–0.47)	0.40 (0.33–0.50)	1.74 (1.39–2.178)	1.85 (1.46–2.35)
NIHSS>/=10	0.27 (0.22–0.33)	0.29 (0.23–0.36)	3.67 (2.95–4.56)	4.99 (4.00–6.22)
IVT	2.42 (2.240–2.61)	2.27 (2.10–2.45)	0.78 (0.70–0.88)	0.72 (0.64–0.80)

*mRS* modified Rankin scale, *EST* endovascular stroke treatment, *IVT* intravenous thrombolysis

For the endpoint ‘decision for palliative care during the hospital treatment’ the regression analysis showed a significant lower proportion in patients treated with EST (OR 0.87; 95% CI 0.78–0.98; p = 0.018) when adjusted for mRS at admission, additive IVT, age, and NIHSS. However, with inclusion of the vessel status at admission into the regression-model, EST was no longer a significant factor.

Details of the regression analyses can be found in Table 3.

## Discussion

In this 2-year observational, retrospective study of a large, country-wide mandatory stroke registry we could demonstrate that the positive effect of endovascular stroke treatment can be demonstrated in an unselected area-wide cohort of ischemic stroke patients. Several studies have shown that EST is applicable in routine clinical practice and has a significant benefit for acute stroke patients with large vessel occlusion.. The patients presented here were on average 10 years older as the patients in the HERMES group [7], had a median NIHSS of 15, and received additional IVT in a significantly lower proportion. These differences in baseline data may partly explain the lower benefit in our cohort (mRS 0–2: OR 1.57).

The SITS-Most Study which also used routine data from many different sites included patients who were on average 5 years younger than ours, had a median NIHSS score of 16 and received IVT in 62% of cases [12]. This analysis, however, did not compare its results with the outcome of non-EST patients. It is reported that 45.5% of patients had a mRS of 0–2 at 90 days. Data from 23 centers were presented from the German Stroke Registry (GSR). These patients were on average 75 years old, had a median NIHSS of 15, and received systemic thrombolysis in 56% of cases. Again, no comparator patients were included. The proportion of patients with a mRS of 0–2 at three months was 37% [13].

In contrast to these data, the study presented here relies on a legally mandatory data collection of all stroke patients and thus also allows the comparison between patients with or without endovascular stroke therapy using statistic adjustments. During the study period almost one out of ten patients with an ischemic stroke was treated with EST in Baden–Wuerttemberg. From the HERMES-RCTs it is not possible to estimate the proportions of patients included in these trials, because no eligibility screening logs were kept. Tsivgoulis et al. estimated that approximately 5 to 8% consecutive acute ischemic stroke patients may be eligible for MT [14] and Hacke and Grotta expected that 5–10% of the patients in large referral centers might be candidates for EST [15]. In Germany, an IVT rate of 16.4% and an EST rate of 6.5% were determined for 2018, with significant differences found between urban regions (EST rate 7.5%) and rural regions (5.3%) [16]. Thus, we believe that we have analyzed a representative cohort of stroke patients.

Our data show that with an appropriate organization of stroke care, successful endovascular stroke therapy is possible in a large country. It requires clear concepts how suitable patients are presented to a thrombectomy center as soon as possible. The cooperation of stroke units of different quality levels has been part of the Baden–Württemberg stroke care concept from the beginning. A survey from 2020 revealed that 13 stroke centers in Baden–Württemberg provide EST 24/7. Of 20 regional stroke units, one offers endovascular stroke therapy 24/7, three others perform thrombectomy during daytime hours (sunshine-EST), and three others are at least partially able to perform endovascular stroke therapy by physicians from neighboring hospitals (ship the doc [17]). Of 18 local stroke units, one provides EST, while the others transfer their patients to neighboring thrombectomy centers. The thrombectomy-centers receive patients from between one and nine enclosed collaborating stroke units. Most local and regional stroke units have clear assignment systems. In addition, in some regions, SOPs in collaboration with emergency services define which patients should be taken directly to a thrombectomy center rather than to the nearer smaller stroke unit, which provides IVT only (mothership vs. drip-and-ship). The data analyzed here, however, were collected for quality assurance purposes. The database does not record the location of the stroke event or the exact place of residence of the patients (for data protection reasons), hence it cannot be traced which is the closest stroke unit. Therefore, our data are not detailed enough to determine which of the above-mentioned care concepts is optimal under which circumstances for which patient.

In addition to EST, IVT was also beneficial for any endpoint in our regression model, both for clinical outcome including mortality, and for the proportion of patients assigned for early palliative therapy. One possible explanation for the observation that the OR for IVT is rather high (e.g. 2.53 for the endpoint mRS 0–1 at discharge) is the 24/7 availability of IVT in any stroke unit in our country. Thus, many patients suitable for IVT can be treated timely, which is still not the case for EVT. In our opinion, this observation also supports the current recommendations to perform endovascular stroke therapy together with systemic thrombolysis in case of an existing indication [18].

A strength of this analysis is the comprehensive data set with more than 38,000 patients, and the connection of the data collection to administrative data, so that the completeness of data is ensured. In the course of two years more than 3820 EST patients were collected from 56 hospitals, while the German Stroke Registry recorded 4340 patients from 25 centers in 4.5 years [19].

Several limitations may mitigate our results: (1) The data quality of the quality control register depends on the accuracy of the information provided by the local center. These data are reviewed with respect to several quality control aims annually, however, source data verification is done only in a very small proportion. (2) Our database was introduced for the purpose of quality assurance and therefore cannot provide all parameters necessary for scientific analyses. For example, neuroradiological details such as infarct sizes, infarct localization, or even just the side of the stroke are not reported in the database. Thus, several prognostic relevant parameters cannot be used for statistical adjustment. (3) There are no long-term follow-up data in this registry, so that data at the time of discharge from hospital treatment, i.e., before the start of outpatient or inpatient rehabilitation, must be used. However, it has been shown that these data already provide a good approximation of the clinical outcome [20]. (4) The data coding has been modified over the years. Therefor it was not possible to include date from more recent years.

## Conclusion

With the use of a large comprehensive stroke dataset, initiated for quality assurance, we could show the benefit of endovascular stroke treatment in the third largest German federal state with more than 11 million inhabitants and sometimes long distances to a thrombectomy center. We also found hints that combination therapy with intravenous thrombolysis is also beneficial.

## Data Availability

Due to legal requirements for the BW-stroke registry data cannot be transferred to third parties. On reasonable request additional analysis are possible.
